# Antiproliferative and Proapoptotic Effects of Chetomin in Human Melanoma Cells

**DOI:** 10.3390/ijms26199835

**Published:** 2025-10-09

**Authors:** Laura Jonderko, Anna Choromańska

**Affiliations:** Department of Molecular and Cellular Biology, Faculty of Pharmacy, Wroclaw Medical University, Borowska 211, 50-556 Wroclaw, Poland; laura.jonderko@student.umw.edu.pl

**Keywords:** chetomin, natural product, fungal metabolite, melanoma, apoptosis, cell proliferation, A375 cells

## Abstract

Melanoma is an aggressive malignancy with poor prognosis in advanced stages, and current therapeutic options provide only limited benefits, highlighting the need for novel treatments. Chetomin, a fungal metabolite isolated from *Chaetomium cochliodes*, has been reported to exhibit diverse biological activities, yet its effects on melanoma cells remain poorly understood. In this study, we evaluated the antitumor potential of chetomin using the human A375 melanoma cell line. Cell viability was assessed with MTT and CellTiter-Glo^®^ assays, which revealed a significant dose- and time-dependent reduction in proliferation following chetomin exposure. Apoptotic effects were confirmed through Annexin V staining, and immunocytochemical analysis demonstrated a concentration-dependent increase in cleaved PARP1, indicating activation of programmed cell death pathways. Collectively, these findings demonstrate that chetomin effectively inhibits melanoma cell growth and promotes apoptosis. The results suggest that chetomin represents a promising lead compound for melanoma therapy, warranting further investigation into its precise molecular mechanisms.

## 1. Introduction

Melanoma is the most aggressive and deadly form of skin cancer, arising from melanocytes, pigment-producing cells responsible for melanin synthesis. It exhibits rapid progression, high metastatic potential, and resistance to various therapies, posing a significant public health challenge [1]. The main factor is ultraviolet (UV) radiation-induced DNA damage, although there are additional risk factors. A common molecular alteration in melanoma is the BRAF V600E mutation, which promotes tumor progression [2]. About 10% of cases are associated with inherited mutations, particularly in the CDKN2A gene, which encodes the tumor suppressors p16^INK4A and p14^ARF [1]. Obesity is also implicated, with increased levels of insulin, IGF-1, and leptin, as well as pro-inflammatory adipokines, contributing to tumor development. Other risk factors include fair skin, light hair, and numerous nevi [3]. Melanoma pathogenesis involves dysregulation of key signaling pathways, notably MAPK and PI3K/AKT, which drive proliferation and inhibit apoptosis [4]. Clinically, early detection and surgical excision are critical for favorable outcomes. Advanced melanoma is treated with immunotherapies, particularly checkpoint inhibitors (anti-PD-1, anti-CTLA-4), and targeted therapies such as BRAF and MEK inhibitors [5,6]. However, treatment resistance and toxicity remain significant issues, emphasizing the need for novel approaches.

**Figure 1:**
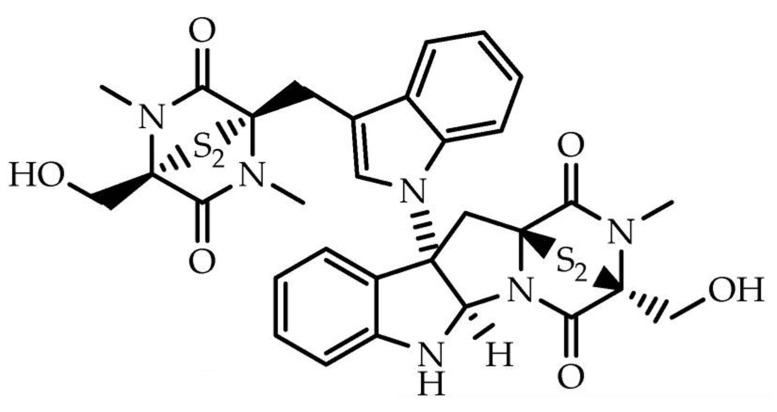
Structures of chetomin [9].

Chetomin is a small-molecule natural product produced by *Chaetomium* species fungi. It was originally reported in 1944 by Waksman and colleagues as a mixture of antibiotics [7]. Initially, chetomin was studied for its antifungal and antibiotic properties and its planar C3-N1′ bond was unknown until the late 1970s [8]. Chetomin belongs to a class of alkaloids known as epidithiodiketopiperazines (ETPs), characterized by a diketopiperazine core and an unusual disulfide bridge (Figure 1).

The disulfide groups in ETPs enable reactivity with thiols or disulfides on cell surfaces, facilitating the formation of stable covalent bonds with transport proteins and subsequent cellular uptake. The intracellular residue enables these alkaloids to participate in various physiological processes, such as the production of reactive oxygen species (ROS), the release of zinc ions (Zn^2+^) or the formation of bonds with cysteine residues in proteins [10]. Although widely distributed in microorganisms, ETPs have also been identified in both plant and animal organisms [11]. Fungi, particularly marine and terrestrial species from the genera *Aspergillus* and *Penicillium*, represent the richest sources of these compounds [12,13].

ETPs exhibit selective cytotoxicity toward cancer cells, with minimal effects on normal cells. For instance, NT1721 reduces the viability of cutaneous T-cell lymphoma cells without significantly affecting normal CD4+ T cells. Structurally related piperazone derivatives show lower toxicity in healthy cells compared to cancerous ones [14,15]. Chetomin exhibits potent antiangiogenic behavior. In a rat aortic ring model, it significantly inhibited microvessel formation, confirming its antiangiogenic properties [16]. As a hypoxia-inducible factor (HIF) inhibitor, chetomin sensitized cancer cells to TRAIL-induced apoptosis by promoting XIAP degradation and activating caspases -3, -8, -9, and -10 [17]. Additionally, it reduced pro-inflammatory cytokines (IL-6), angiogenic factors (VEGF), and ischemia-induced tissue damage [17,18]. Chetomin demonstrated strong cytotoxicity against multiple myeloma cell lines by downregulating HIF-1-regulated genes, including CA9 and VEGF [19,20]. It also showed protective effects in lung transplant ischemia–reperfusion injury through HIF-1α inhibition and modulation of ROS production [21]. In triple-negative breast cancer cells, chetomin induced apoptosis via mitochondrial dysfunction, PI3K/mTOR pathway inhibition, ER stress, and calcium overload [22].

Chetomin represents a promising candidate for further preclinical and clinical investigation, including its potential in antiviral therapy. In silico studies have identified chetomin as a potent inhibitor of the 3CLpro protease, a key enzyme involved in SARS-CoV-2 replication, showing high binding stability and favorable pharmacokinetic properties [23]. Its ability to enhance antiviral responses appears to be HIF-dependent. In vitro experiments demonstrated that chetomin increased the sensitivity of 786-O renal cancer cells to VSV-induced cytolysis, indicating its potential to support oncolytic virotherapy [24]. Additionally, chetomin exhibited immunosuppressive activity by inhibiting the proliferation of T and B lymphocytes in the mouse spleen at micromolar concentrations [25].

Most bioactive compounds of natural origin face challenges related to large-scale production. A major limitation in the clinical use of chetomin is its highly hydrophobic nature, which severely impairs its solubility and bioavailability. To address these issues, advanced drug delivery systems such as biodegradable polymeric micelles have been developed to enhance solubility and improve the therapeutic properties of this compound [26]. Despite the growing body of evidence supporting chetomin’s anticancer properties in multiple myeloma, breast cancer, and other malignancies, its activity in melanoma has not been systematically investigated. This gap is particularly important given that melanoma is characterized by strong resistance to apoptosis and high adaptability to hypoxic conditions, in which HIF-1 signaling plays a central role. Since chetomin is a potent HIF inhibitor and has been shown to sensitize other tumor types to apoptotic stimuli, evaluating its effects in melanoma may uncover novel therapeutic opportunities. Moreover, current melanoma therapies are often limited by resistance to BRAF/MEK inhibitors and immune checkpoint blockade, underscoring the need for alternative strategies. Therefore, this study aims to assess the antitumor potential of chetomin in human melanoma cells, focusing on its impact on cell viability and apoptosis.

A375 was selected as a benchmark BRAFV600E-mutant melanoma model that is extensively used in studies of BRAF/MEK-targeted therapies and exhibits robust hypoxia/HIF-1α–related responses, providing mechanistic relevance to our aims and enabling cross-study comparability [2,4,6].

## 2. Results

### 2.1. Chetomin Inhibits Cell Viability of Melanoma Cells

**Figure 2:**
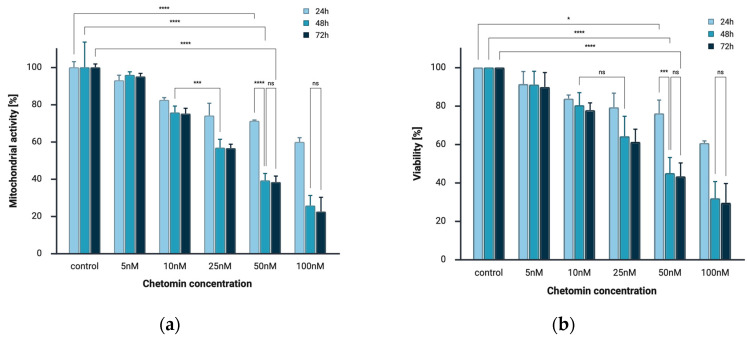
Chetomin decreases the viability of melanoma cells in a concentration- and time-dependent manner. (**a**) A375 cell viability measured by MTT assay after 24, 48, and 72 h of chetomin treatment. (**b**) A375 cell viability determined by CellTiter-Glo^®^ 2.0 assay after 24, 48, and 72 h of treatment. Both assays consistently showed a significant reduction in viability at higher chetomin concentrations and longer exposure times. Data are presented as mean ± SD (*n* = 3 independent experiments). Statistical significance was determined by two-way ANOVA followed by Bonferroni’s post hoc test; * *p* < 0.05, *** *p* < 0.001, **** *p* < 0.0001 vs. control. ns—not significant.

To investigate the effects of chetomin treatment on cell viability, human melanoma cells A375 were treated with various concentrations of chetomin (0, 5, 10, 25, 50, 100 nM) for 24, 48, 72 h. The results of MTT and CellTiter-Glo^®^ 2.0 Cell Viability assays demonstrated that chetomin strongly decreased the viability of melanoma cells in a concentration-dependent and time-dependent manner. In both assays, the strongest cytotoxic effect was observed after 72 h of incubation at the highest applied concentration of chetomin (100 nM), from 100% to 22.5% in the MTT assay and to 36.77% in the CellTiter-Glo^®^ assay (Figure 2).

The most pronounced decrease in cell viability occurred between 24 and 48 h of exposure. Differences between 48 and 72 h were less marked, suggesting that the primary cytotoxic activity of chetomin takes place within the first 48 h of treatment.

### 2.2. Chetomin Induces Apoptosis in the Human Melanoma A375

**Figure 3:**
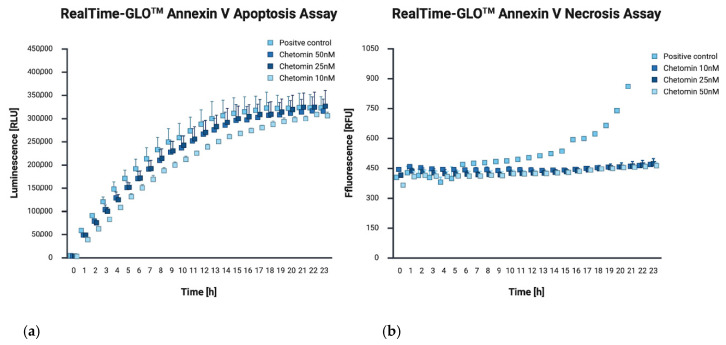
Chetomin induces apoptosis. (**a**) Luminescence measurement expressed in Relative Luminescence Units (RLU) over a 24 h period, corresponding to apoptosis, (**b**) fluorescence measurement expressed in Relative Fluorescence Units (RFU) over a 24 h period, corresponding to necrosis.

The RealTime-Glo™ Annexin V Apoptosis and Necrosis Assay was employed to simultaneously and quantitatively assess two key cellular events associated with cell death: phosphatidylserine (PS) externalization on the outer leaflet of the plasma membrane (a hallmark of apoptosis), and loss of membrane integrity (a marker of necrosis). Based on the luminescence (Annexin V binding) and fluorescence (DNA dye uptake following membrane disruption) curves obtained for three different concentrations of the test compound (10, 25, and 50 nM), a clear and consistent increase in luminescence signal was observed under all conditions. This signal increased over time and reached a plateau after approximately 20–25 h of incubation, indicating the induction of apoptosis, consistent with the mechanism by which Annexin V binds to PS exposed on the cell surface during the early phase of apoptotic cell death. In contrast to the luminescent signal, the fluorescence signal, indicative of compromised membrane integrity, remained low throughout the experiment (Figure 3).

### 2.3. Immunocytochemical Detection of Cleaved PARP1

**Figure 4:**
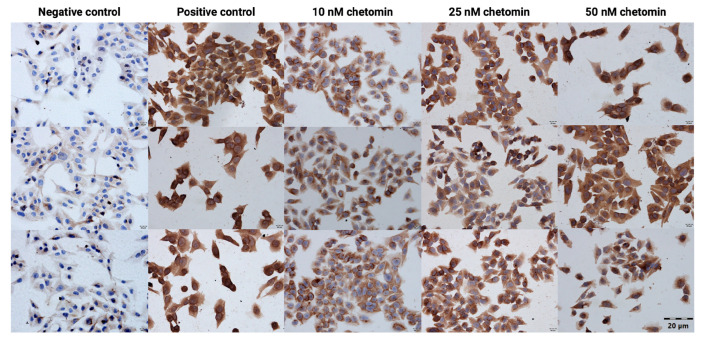
Immunocytochemical detection of cleaved PARP1 in A375 cells treated with increasing concentrations of chetomin (0, 10, 25, and 50 nM) and with the positive control doxorubicin (0.5 μM). Cells were stained using the Mouse and Rabbit Specific HRP/DAB (ABC) Detection IHC Kit. DAB staining (brown) indicates cleaved PARP1 immunoreactivity, and nuclei were counterstained with hematoxylin (blue).

Immunocytochemical analysis revealed minimal cleaved PARP1 immunoreactivity in the negative control group, with nuclei counterstained in blue by hematoxylin. In contrast, the positive control (doxorubicin, 0.5 μM) showed strong, diffuse cytoplasmic and nuclear brown DAB staining, indicating high levels of cleaved PARP1. Chetomin treatment resulted in a concentration-dependent increase in cleaved PARP1 expression (Figure 4 and Table 1).

Our analysis revealed a dose-dependent increase in cleaved PARP1 immunoreactivity, reaching levels comparable to the positive control (0.5 μM doxorubicin) at the highest concentration (50 nM).

## 3. Discussion

The conducted study enabled a comprehensive evaluation of the cytotoxic effects of chetomin on A375 melanoma cells, both in terms of cell viability and the mechanism of induced cell death. To our knowledge, this is the first report documenting the induction of apoptosis by chetomin in this cell line, suggesting its potential as a promising agent in melanoma therapy.

The obtained results demonstrate that chetomin exhibits significant cytotoxic activity against A375 melanoma cells. Both the MTT assay and the CellTiter-Glo^®^ 2.0 assay confirmed that chetomin exerts dose- and time-dependent cytotoxic effects. Mitochondrial activity and ATP levels were significantly reduced at concentrations as low as 10 nM, with the maximal effect observed after 72 h of incubation at 100 nM. The IC_50_ values of ~23 nM, 19 nM, and 21 nM at 24, 48, and 72 h, as determined by the MTT assay. Similar results were obtained using the CellTiter-Glo^®^ assay, yielding IC_50_ values of ~222 nM at 24 h and ~34 nM at both 48 h and 72 h. These findings indicate that melanoma cells are sensitive to chetomin within the low nanomolar range, particularly after prolonged exposure. When compared with other cancer models, the IC_50_ values observed in A375 cells are higher than those reported in multiple myeloma, with median IC_50_ values of 4.1 nM across human myeloma cell lines (Moreaux et al. [27]) and as low as 1.56 nM in primary myeloma cells (Storti et al. [28]). This suggests that multiple myeloma cells are more susceptible to chetomin than melanoma cells. Our findings are consistent with the general sensitivity of solid tumor models to epidithiodiketopiperazine derivatives. The structurally related compound NT1721 displayed nanomolar activity in cutaneous T cell lymphoma, with IC_50_ values ranging from 6 to 125 nM in HuT78 cells and from 70 to 542 nM in HH cells depending on the incubation time (48–72 h) [14,20].

The molecular basis of chetomin-induced cytotoxicity in melanoma cells remains to be fully elucidated, but several mechanisms described in other cancer models may be relevant. Chetomin is a well-established inhibitor of HIF-1 transcriptional activity, and melanoma is known to thrive under hypoxic conditions where HIF-1 signaling promotes angiogenesis, metabolic adaptation, and resistance to apoptosis. Thus, inhibition of HIF-1 by chetomin could contribute to the observed induction of apoptosis in A375 cells. In addition, chetomin has been reported to interfere with the PI3K/mTOR pathway and to trigger mitochondrial dysfunction in breast cancer cells. Given that PI3K/AKT/mTOR signaling is frequently dysregulated in melanoma and plays a key role in cell survival, chetomin-mediated inhibition of this pathway may further enhance apoptotic susceptibility. Although we did not directly investigate these mechanisms in the present study, the observed reduction in ATP levels and the activation of apoptotic markers are consistent with interference in these survival pathways. Future work should therefore focus on dissecting the relative contribution of HIF-1 inhibition and PI3K/mTOR modulation to chetomin’s activity in melanoma.

To contextualize potency, chetomin’s low-nanomolar IC_50_ values in A375 fall within a range that is typically more potent than classical chemotherapeutics used in melanoma (e.g., dacarbazine, platinum agents) yet generally below that of targeted MAPK-pathway inhibitors (e.g., BRAF/MEK inhibitors) reported in A375 under comparable in vitro conditions [2,4,6]. This positioning supports chetomin as a mechanistically interesting candidate that could complement, rather than replace, established targeted therapies, particularly in settings associated with hypoxia/HIF-1α-driven adaptation.

Application of the RealTime-Glo™ Annexin V Apoptosis and Necrosis Assay allowed for a detailed analysis of the mechanism underlying chetomin-induced cell death. The results showed that apoptosis is the predominant mode of action, as evidenced by a rapid and pronounced increase in luminescence signal, corresponding to Annexin V binding to phosphatidylserine. In contrast, the fluorescence signal indicative of membrane integrity loss remained low during the observation period, suggesting that early secondary necrosis was not a major contributor to cell death. The immunocytochemical detection of cleaved PARP1 further supported the pro-apoptotic activity of chetomin in A375 cells. The concentration-dependent increase in brown DAB staining intensity, particularly evident at 25 nM and 50 nM, confirmed the activation of the apoptotic cascade at the molecular level. This observation is consistent with the Annexin V assay results, indicating early apoptotic changes, and highlights PARP1 cleavage as a downstream event in chetomin-induced cell death. It should be noted, however, that both the Annexin V assay and PARP1 immunocytochemistry primarily captured early apoptotic events within the first 24 h of treatment. Considering that cell viability declined to ~22.5% after 72 h, it is highly likely that a considerable fraction of cells subsequently progressed to late apoptosis and secondary necrosis. Since we did not directly quantify apoptosis and necrosis at these later time points, our conclusions regarding the absence of secondary necrosis are limited to the early phase of chetomin-induced cell death. Future studies with extended time-course analyses will be necessary to fully characterize the dynamics of apoptosis-to-necrosis transition in melanoma cells exposed to chetomin.

Overall, these findings indicate that chetomin possesses anticancer potential and may serve as a promising candidate for further investigation in the context of melanoma treatment.

## 4. Materials and Methods

### 4.1. Cell Culture and Reagents

The investigation was performed using the human melanoma cell line A375 (ATCC^®^ CRL-1619™, LGC Standards, Kiełpin, Poland), originally derived from the skin of a 54-year-old female patient. Cells were maintained as adherent monolayers in 75 mL polystyrene culture flasks (Falcon^®^, Corning Life Sciences, Tewksbury, MA, USA). They were propagated in Dulbecco’s modified Eagle’s medium (DMEM; Sigma-Aldrich, St. Louis, MO, USA) enriched with 10% fetal bovine serum and a standard antibiotic mixture (penicillin/streptomycin). The cells were incubated at 37 °C in a humidified atmosphere with 5% CO_2_ (Hereus^®^, Thermo Fisher Scientific, Waltham, MA, USA). Routine subculturing was carried out using trypsin/EDTA (0.025% trypsin and 0.02% EDTA; Sigma-Aldrich), and the absence of Mycoplasma contamination was regularly confirmed. A375 (ATCC^®^ CRL-1619™) harbors the BRAFV600E mutation and is widely used as a reference line in melanoma pharmacology, particularly in studies of MAPK pathway inhibition [2,4].

### 4.2. Compound and Drug Preparation

Chetomin (NSC 289491, sourced from Chaetomium cochliodes, ≥98% purity as determined by HPLC) was dissolved in dimethyl sulfoxide (DMSO) to prepare a stock solution with a final concentration of 100 nM. This stock solution was further diluted in culture medium to obtain the working concentrations used in the experiments.

### 4.3. In Vitro Cytotoxicity and Antiproliferative Activity Assessment

#### 4.3.1. MTT Assay

The cells’ viability was determined by assessing their mitochondrial activity. After cells were trypsinized, A375 cells were exposed to chetomin at final concentrations of 5, 10, 25, 50, or 100 nM and seeded into 96-well plates (3 × 10^4^ cells/well). Viability was assessed after 24, 48, and 72 h according to the manufacturer’s standard protocol. The culture medium was removed from each well, replaced with 100 µL of MTT solution (0.5 mg/mL in PBS; Sigma-Aldrich), and cells were incubated for 2 h at 37 °C to allow formazan crystal formation. The crystals were subsequently solubilized in acidified isopropanol (0.04 M HCl in 99.9% isopropanol, 100 µL/well) by pipetting. The absorbance of each well was measured at 570 nM using a multi-plate reader (GloMax, Promega, Walldorf, Germany), and results were normalized to the untreated control group.

#### 4.3.2. CellTiter-Glo^®^ 2.0 Cell Viability Assay

Cell viability was measured using the CellTiter-Glo^®^ 2.0 Cell Viability Assay (Promega, Madison, WI, USA). A375 cells were trypsinized, counted, and seeded into opaque, white 96-well plates at a density of 3 × 10^4^ cells per well in complete culture medium (100 µL/well), then incubated overnight (≈16–18 h) to allow attachment. The next day, the medium was replaced with fresh medium containing chetomin at final concentrations of 5, 10, 25, 50, or 100 nM. Chetomin was added from a concentrated DMSO stock; the final DMSO content was kept constant across all wells (including vehicle controls). Plates were incubated for 24, 48, or 72 h under standard culture conditions (37 °C, 5% CO_2_). Following incubation, plates were equilibrated to room temperature for ~30 min. An equal volume of CellTiter-Glo^®^ 2.0 reagent was added to each well (100 µL reagent to 100 µL medium), plates were shaken for 2 min on an orbital shaker to induce cell lysis, and then incubated for 10 min at room temperature to stabilize the luminescent signal. Luminescence was measured using a GloMax^®^ multi-mode plate reader.

### 4.4. Apoptosis Analysis

Apoptotic and necrotic cell death in A375 melanoma cells exposed to chetomin was assessed using the RealTime-Glo™ Annexin V Apoptosis and Necrosis Assay (Promega, Madison, WI, USA). This method permits simultaneous, real-time assessment of apoptotic and necrotic processes in viable cultures. Cells were seeded in white 96-well plates (3 × 10^4^ cells/well) and allowed to adhere overnight under standard culture conditions. Chetomin was added at final concentrations of 10, 25, or 50 nM, and assay reagents were supplied according to the manufacturer’s instructions, in the following order: Annexin V NanoBiT^®^ Substrate, CaCl_2_, Necrosis Detection Reagent, Annexin V-SmBiT Reagent, and Annexin V-LgBiT Reagent. Luminescent and fluorescent readouts, corresponding to apoptosis and necrosis, respectively, were recorded for up to 24 h using a GloMax^®^ plate reader.

### 4.5. Detection of Cleaved PARP1

To evaluate apoptosis induction, immunocytochemical staining for cleaved PARP1 was performed on A375 melanoma cells treated with chetomin at final concentrations of 0, 10, 25, and 50 nM for 24 h, as well as with the positive control doxorubicin (0.5 μM). Cells were fixed in 4% paraformaldehyde for 15 min at room temperature, washed with PBS, and permeabilized with 0.1% Triton X-100. Endogenous peroxidase activity was quenched with 3% hydrogen peroxide. The samples were then incubated overnight at 4 °C with a primary antibody against cleaved PARP1 (rabbit monoclonal antibody [E51], Abcam, Cambridge, UK; catalog no. ab32064) at a dilution of 1:100. Detection was carried out using the Mouse and Rabbit Specific HRP/DAB (ABC) Detection IHC Kit (ab64264, Abcam, UK) according to the manufacturer’s protocol. Briefly, slides were incubated with the secondary antibody, followed by the streptavidin-HRP complex, and visualized using 3,3′-diaminobenzidine (DAB) as chromogen. Counterstaining was performed with hematoxylin, and slides were mounted in aqueous mounting medium. Staining was assessed using a light microscope.

### 4.6. Statistical Analysis

Data represent mean ± standard deviation (SD) from three independent experiments. Statistical significance was determined using two-way ANOVA (treatment concentration × incubation time), followed by Bonferroni’s post hoc test.

## Figures and Tables

**Table 1:** Immunocytochemical evaluation of cleaved PARP1 expression in A375 cells treated with chetomin. The evaluation of stained reaction: (−) negative, no reaction; (+) weak, (++) moderate, and (+++) strong.

Condition	Intensity of Staining	Positively Stained Cells [%]
Negative control	-	0
Positive control	+++	100
10 nM chetomin	+	100
25 nM chetomin	++	100
50 nM chetomin	+++	100

## Data Availability

The data presented in this study are available on request from the corresponding author.

## Abbreviations

ATP	Adenosine Triphosphate
BRAF	v-Raf murine sarcoma viral oncogene homolog B
CDKN2A	Cyclin-Dependent Kinase Inhibitor 2A
CTLA-4	Cytotoxic T-Lymphocyte-Associated Protein 4
DAB	3,3′-Diaminobenzidine
DMEM	Dulbecco’s Modified Eagle Medium
DMSO	Dimethyl sulfoxide
EDTA	Ethylenediaminetetraacetic Acid
ETP	Epidithiodiketopiperazine
FBS	Fetal Bovine Serum
HIF	Hypoxia-Inducible Factor
HRP	Horseradish Peroxidase
IHC	Immunohistochemistry
IGF-1	Insulin-Like Growth Factor 1
MAPK	Mitogen-Activated Protein Kinase
MEK	MAPK/ERK Kinase
mTOR	Mammalian Target of Rapamycin
MTT	3-(4,5-Dimethylthiazol-2-yl)-2,5-Diphenyltetrazolium Bromide
PARP1	Poly (ADP-Ribose) Polymerase 1
PBS	Phosphate-Buffered Saline
PD-1	Programmed Cell Death Protein 1
PI3K	Phosphoinositide 3-Kinase
PS	Phosphatidylserine
ROS	Reactive Oxygen Species
SARS-CoV-2	Severe Acute Respiratory Syndrome Coronavirus 2
SD	Standard Deviation
VEGF	Vascular Endothelial Growth Factor
VSV	Vesicular Stomatitis Virus
XIAP	X-Linked Inhibitor of Apoptosis Protein

## References

[1] Slominski R.M., Kim T.-K., Janjetovic Z., Brożyna A.A., Podgorska E., Dixon K.M., Mason R.S., Tuckey R.C., Sharma R., Crossman D.K. (2024). Malignant Melanoma: An Overview, New Perspectives, and Vitamin D Signaling. Cancers.

[2] Frech F.S., Bommareddy K., Hernandez L., Dreyfuss I., Urbonas R., Nouri K. (2022). Cutaneous Melanoma: An Update on Pathogenesis, Prevention, and Treatment. Dermatol. Rev..

[3] Szostak A.P., Szopińska K., Graca M., Śmigielska-Mikołajczyk M.J., Łowicka W., Dyląg L., Wawszkowicz K., Oluszczak K., Szeliga A., Korta K. (2024). Prevention and Early Detection of the Most Aggressive Skin Cancer: Melanoma. Qual. Sport.

[4] Kim H.J., Kim Y.H. (2024). Molecular Frontiers in Melanoma: Pathogenesis, Diagnosis, and Therapeutic Advances. Int. J. Mol. Sci..

[5] Siegel R.L., Miller K.D., Jemal A. (2016). Cancer Statistics, 2016. CA. Cancer J. Clin..

[6] Hsieh M.-Y., Hsu S.-K., Liu T.-Y., Wu C.-Y., Chiu C.-C. (2024). Melanoma Biology and Treatment: A Review of Novel Regulated Cell Death-Based Approaches. Cancer Cell Int..

[7] Geiger W.B. (1949). Chetomin an Antibiotic Substance from Chaetomium Cochliodes; Composition and Functional Groups. Arch. Biochem..

[8] McInnes A.G., Taylor A., Walter J.A. The Structure of Chetomin. https://pubs.acs.org/doi/pdf/10.1021/ja00437a074.

[9] Gomes N.G.M., Pereira R.B., Andrade P.B., Valentão P. (2019). Double the Chemistry, Double the Fun: Structural Diversity and Biological Activity of Marine-Derived Diketopiperazine Dimers. Mar. Drugs.

[10] Wang L., Jiang Q., Chen S., Wang S., Lu J., Gao X., Zhang D., Jin X. (2023). Natural Epidithiodiketopiperazine Alkaloids as Potential Anticancer Agents: Recent Mechanisms of Action, Structural Modification, and Synthetic Strategies. Bioorganic Chem..

[11] Borthwick A.D. (2012). 2,5-Diketopiperazines: Synthesis, Reactions, Medicinal Chemistry, and Bioactive Natural Products. Chem. Rev..

[12] Ma Y.-M., Liang X.-A., Kong Y., Jia B. (2016). Structural Diversity and Biological Activities of Indole Diketopiperazine Alkaloids from Fungi. J. Agric. Food Chem..

[13] Li S.-M. (2010). Prenylated Indole Derivatives from Fungi: Structure Diversity, Biological Activities, Biosynthesis and Chemoenzymatic Synthesis. Nat. Prod. Rep..

[14] Lin M., Kowolik C.M., Xie J., Yadav S., Overman L.E., Horne D.A. (2021). Potent Anticancer Effects of Epidithiodiketopiperazine NT1721 in Cutaneous T Cell Lymphoma. Cancers.

[15] Ghasemi S., Sharifi S., Shahbazi Mojarrad J. (2020). Design, Synthesis and Biological Evaluation of Novel Piperazinone Derivatives as Cytotoxic Agents. Adv. Pharm. Bull..

[16] Reece K.M., Richardson E.D., Cook K.M., Campbell T.J., Pisle S.T., Holly A.J., Venzon D.J., Liewehr D.J., Chau C.H., Price D.K. (2014). Epidithiodiketopiperazines (ETPs) Exhibit in Vitro Antiangiogenic and in Vivo Antitumor Activity by Disrupting the HIF-1α/P300 Complex in a Preclinical Model of Prostate Cancer. Mol. Cancer.

[17] Yano K., Horinaka M., Yoshida T., Yasuda T., Taniguchi H., Goda A.E., Wakada M., Yoshikawa S., Nakamura T., Kawauchi A. (2011). Chetomin Induces Degradation of XIAP and Enhances TRAIL Sensitivity in Urogenital Cancer Cells. Int. J. Oncol..

[18] Bravo-Reyna C.C., Miranda-Galván V., Reyes-Soto G., Vicuña R., Alanis-Mendizabal J., Escobar-Valderrama M., Arango D., Bautista C.J., Ramírez V., Torres-Villalobos G. (2025). Evaluation of the Chetomin Effect on Histopathological Features in a Murine Acute Spinal Cord Injury Model. World Neurosurg. X.

[19] Viziteu E., Grandmougin C., Goldschmidt H., Seckinger A., Hose D., Klein B., Moreaux J. (2016). Chetomin, Targeting HIF-1α/P300 Complex, Exhibits Antitumour Activity in Multiple Myeloma. Br. J. Cancer.

[20] Staab A., Loeffler J., Said H.M., Diehlmann D., Katzer A., Beyer M., Fleischer M., Schwab F., Baier K., Einsele H. (2007). Effects of HIF-1 Inhibition by Chetomin on Hypoxia-Related Transcription and Radiosensitivity in HT 1080 Human Fibrosarcoma Cells. BMC Cancer.

[21] Bravo-Reyna C.C., Zentella A., Ventura-Gallegos J.L., Torres-Villalobos G., Miranda-Galván V., Alanis-Mendizabal J., Escobar-Valderrama J.M., Nava C., Díaz-Martínez N.E., Bliskunova T. (2024). Experimental Lung Transplantation Related with HIF-1, VEGF, ROS. Assessment of HIF-1α, VEGF, and Reactive Oxygen Species after Competitive Blockade of Chetomin for Lung Transplantation in Rats. Physiol. Res..

[22] Dewangan J., Srivastava S., Mishra S., Pandey P.K., Divakar A., Rath S.K. (2018). Chetomin Induces Apoptosis in Human Triple-Negative Breast Cancer Cells by Promoting Calcium Overload and Mitochondrial Dysfunction. Biochem. Biophys. Res. Commun..

[23] Ibrahim M.A.A., Abdelrahman A.H.M., Mohamed D.E.M., Abdeljawaad K.A.A., Naeem M.A., Gabr G.A., Shawky A.M., Soliman M.E.S., Sidhom P.A., Paré P.W. (2023). Chetomin, a SARS-CoV-2 3C-like Protease (3CLpro) Inhibitor: In Silico Screening, Enzyme Docking, Molecular Dynamics and Pharmacokinetics Analysis. Viruses.

[24] Hwang I.I.L., Watson I.R., Der S.D., Ohh M. (2006). Loss of VHL Confers Hypoxia-Inducible Factor (HIF)-Dependent Resistance to Vesicular Stomatitis Virus: Role of HIF in Antiviral Response. J. Virol..

[25] Fujimoto H., Sumino M., Okuyama E., Ishibashi M. (2004). Immunomodulatory Constituents from an Ascomycete, Chaetomium Seminudum. J. Nat. Prod..

[26] Wu Q., Li G., Deng S., Ouyang L., Li L., Liu L., Luo N., Song X., He G., Gong C. (2014). Enhanced Antitumor Activity and Mechanism of Biodegradable Polymeric Micelles-Encapsulated Chetomin in Both Transgenic Zebrafish and Mouse Models. Nanoscale.

[27] Moreaux J., Klein B., Bataille R., Descamps G., Maïga S., Hose D., Goldschmidt H., Jauch A., Rème T., Jourdan M. (2011). A high-risk signature for patients with multiple myeloma established from the molecular classification of human myeloma cell lines. Haematologica.

[28] Storti P., Bolzoni M., Donofrio G., Airoldi I., Guasco D., Toscani D., Martella E., Lazzaretti M., Mancini C., Agnelli L. (2013). Hypoxia-inducible factor (HIF)-1α suppression in myeloma cells blocks tumoral growth in vivo inhibiting angiogenesis and bone destruction. Leukemia.

